# Editorial of Special Issue “The Role of Vitamin D in Human Health and Diseases 2.0”

**DOI:** 10.3390/ijms24054337

**Published:** 2023-02-22

**Authors:** Francesca Silvagno, Loredana Bergandi

**Affiliations:** Department of Oncology, University of Torino, Via Santena 5 bis, 10126 Torino, Italy

Vitamin D performs a differentiating, metabolic and anti-inflammatory function, through genomic, non-genomic and mitochondrial mechanisms of action. In fact, vitamin D not only controls the transcription of thousands of genes and is involved in the modulation of calcium fluxes, but it also influences cellular metabolism and the maintenance of specific nuclear programs. Due to its broad spectrum of action, and because of the large number of target tissues, the deficiency of vitamin D and the molecular defects of its signaling pathway, such as VDR polymorphism or epigenetic silencing, can be involved in many pathologies.

In this Special Issue, many scientists have contributed to elucidating the role of vitamin D in health and disease. Eight research articles and a compendium of nine reviews are published, covering different aspects of the activity of vitamin D and its defective signaling or low levels associated with many pathological conditions. Among diseases, the impact of vitamin D deficiency and supplementation in COVID-19 remains a hot topic, as revealed in the previous Special Issue “The role of vitamin D in human health and diseases” and confirmed in this collection. Here, in addition to a systematic review discussing the evidence of beneficial effects of vitamin D supplementation in COVID-19 patients [1], two additional studies focused on the implications of vitamin D deficiency in the COVID-19-related acute kidney injury (AKI), which is one of the most common extrapulmonary manifestations of this infectious disease. The review from Hsieh et al. highlighted the cross-relationship between vitamin D deficiency and AKI, reporting the evidence of their mutual causality and the molecular mechanisms triggered by SARS-CoV-2 infection in inducing both dysfunctions [2]. Similarly, a narrative review from Liao et al. explored the potential role of vitamin D in preventing AKI in COVID-19 patients, but with a focus on the urokinase-type plasminogen activator/soluble urokinase-type plasminogen activator receptor (uPA/suPAR) pathway and its possible relationship with vitamin D in patients with COVID-19-related AKI [3]. Moreover, the role of vitamin D deficiency in COVID-19 was analyzed by interrogating genetic and biological databases. Genome-wide association studies were explored by enrichment analyses of the pathways, in search of gene-disease associations. Although the study reported the absence of a common genetic background between vitamin D deficiency and COVID-19, it supported the possibility that a vitamin D deficiency causes comorbid conditions linked to a higher risk of developing severe COVID-19 disease [4]. Furthermore, one study investigated the relationship between vitamin D deficiency in pregnant women and the severity of COVID-19 infection. Low vitamin D levels were detected in symptomatic, but not asymptomatic, COVID-19 patients, and vitamin D deficiency was described as an independent predictor of severe COVID-19, supporting the recommendation of vitamin D supplementation during pregnancy to avoid worse COVID-19 outcomes [5]. 

The effect of vitamin D in pregnancy was considered in two other studies of this collection. A systematic review examined the associations between maternal vitamin D levels and offspring neuropsychiatric and psychiatric outcomes, finding a positive association for attention-deficit/hyperactivity disorder and schizophrenia [6]. Moreover, a research article investigated the role of chorionic somatomammotropin hormone (CSH) in the regulation of calcium, phosphate and vitamin D utilization in late gestation of sheep. This study provided a model of the hormonal regulation of mineral transport during pregnancy from which further understanding in mammalian species can be achieved [7]. 

Among the signaling pathways analyzed in this collection, the antioxidant and anti-inflammatory properties of vitamin D were largely investigated, and several studies revealed the central role of vitamin D in inflammatory diseases, such as COVID-19, diabetes, AKI, liver and kidney disease. The anti-inflammatory effects of vitamin D were explored in the adipose tissue of obese mice fed a high-fat diet [8], in T cell immunity [9] and in the analysis of inflammatory markers in healthy Saudi males [10]. All studies demonstrated that the treatment with vitamin D significantly reduced the level of pro-inflammatory cytokines, measured in serum [10] or in activated T cells [9]. Interesting experimental work carried out on obese mice revealed that the beneficial effects of the hormone were detected as decreased levels of pro-inflammatory cytokines, attenuated obesity-induced adipose hypertrophy and macrophage recruitment, and were related to increased AMPK activity and suppressed NF-κB phosphorylation [8]. Vitamin D deficiency correlates with the severity of many pathologies, for example, in Type 1 diabetes, as reported by a systematic review [11], and in chronic kidney disease and kidney transplant [12]. The reduced levels of vitamin D also correlate with chronic liver disease and have a predictive role in complications and the progression of advanced disease [13]. A combination of poor vitamin D status and dysbiosis may contribute to the progression of cardiometabolic diseases, as can be inferred from the accurate review that presents the relationship among vitamin D, microbiota and cardiometabolic diseases, with a focus on metabolic syndrome [14]. 

Among the causes of variability in vitamin D signaling, single nucleotide polymorphisms (SNPs) are to be mentioned. Indeed, SNPs can influence the expression and/or functions of the VDR and of other genes in the vitamin D metabolic pathways, and may justify interindividual differences in responsiveness to the hormone. One study of this Special Issue explored five SNPs in the VDR gene in order to unravel the role of genetic polymorphisms on VDR expression in periodontal fibroblasts during simulated orthodontic compressive force [15]. Genetic polymorphisms were also investigated in the systematic review by Jaroenlapnopparat et al. [16], which revealed the association between the variations of several genes in the vitamin D metabolic pathway and the occurrence, severity and response to the treatment of non-alcoholic fatty liver disease (NAFLD). These findings support the notion that the vitamin D signaling pathway may play a significant role in the pathogenesis of NAFLD, in agreement with the reported beneficial effects of vitamin D supplementation described in the published review by Ravaioli et al. [13].

Finally, the effects of vitamin D supplementation were investigated in inflammatory diseases [8,10] and in metabolism [17]. The latter was analyzed in a randomized crossover study carried out in post-menopausal women. The findings of this trial revealed an influence of diurnal rhythm on the plasma metabolome, while vitamin D supplementation appeared to have little influence on fluctuations in the plasma metabolome.

As a whole, this Special Issue contains an interesting combination of research articles and reviews that present novel data on the effects of vitamin D in many pathologies, such as COVID-19 and diabetes, and in kidney, liver and cardiometabolic disease. The anti-inflammatory properties of vitamin D are the “fil rouge” in the heterogeneous spectrum of beneficial effects of the hormone described in this collection, which discusses current knowledge of the impact of vitamin D deficiency and supplementation in a variety of diseases.
